# Reporting Multiple Individual Injuries in Studies of Team Ball Sports: A Systematic Review of Current Practice

**DOI:** 10.1007/s40279-016-0637-3

**Published:** 2016-10-26

**Authors:** Lauren V. Fortington, Henk van der Worp, Inge van den Akker-Scheek, Caroline F. Finch

**Affiliations:** 1 Australian Collaboration for Research into Injury in Sport and its Prevention (ACRISP), Federation University Australia, SMB Campus, PO Box 663, Ballarat, VIC 3353 Australia; 2 Center for Sports Medicine, University of Groningen, University Medical Center Groningen, Groningen, The Netherlands

## Abstract

**Background:**

To identify and prioritise targets for injury prevention efforts, injury incidence studies are widely reported. The accuracy and consistency in calculation and reporting of injury incidence is crucial. Many individuals experience more than one injury but multiple injuries are not consistently reported in sport injury incidence studies.

**Objective:**

The aim of this systematic review was to evaluate current practice of how multiple injuries within individuals have been defined and reported in prospective, long-term, injury studies in team ball sports.

**Data Sources:**

A systematic search of three online databases for articles published before 2016.

**Study Selection:**

Publications were included if (1) they collected prospective data on musculoskeletal injuries in individual participants; (2) the study duration was >1 consecutive calendar year/season; and (3) individuals were the unit of analysis.

**Data Extraction:**

Key study features were summarised, including definitions of injury, how multiple individual injuries were reported and results relating to multiple injuries.

**Results:**

Of the 71 publications included, half did not specifically indicate multiple individual injuries; those that did were largely limited to reporting recurrent injuries. Eight studies reported the number/proportion of athletes with more than one injury, and 11 studies presented the mean/number of injuries per athlete.

**Conclusions:**

Despite it being relatively common to collect data on individuals across more than one season, the reporting of multiple injuries within individuals is much more limited. Ultimately, better addressing of multiple injuries will improve the accuracy of injury incidence studies and enable more precise targeting and monitoring of the effectiveness of preventive interventions.

**Electronic supplementary material:**

The online version of this article (doi:10.1007/s40279-016-0637-3) contains supplementary material, which is available to authorized users.

## Key Points

**Table:** 

While there is an increasing awareness of and increasing number of publications that report the collection of individual injury data across more than one season/year, the reporting of this injury data appears to be challenging.
Half of the publications identified reported the total number of injuries or injured athletes as an overall grouped result across the entire study duration.
Studies that recognised multiple individual injuries were largely limited to reporting recurrent injuries (of the exact same type and side).
Injury prevention efforts rely on accurate incidence estimates, and ongoing developments toward better reporting of multiple injuries is encouraged.

## Introduction

For people who participate in team ball sports, injuries can unfortunately be a common occurrence. Reducing the chance of sustaining an injury is of importance for athletes, team support staff (e.g. coaches and trainers) and sports/health bodies, not only for individual health protection but also for broader benefits, such as better team performance [1, 2] and encouraging continued participation in sport [3]. Investigation of injury incidence is the basis of injury prevention as it is needed to identify the sports, injury types or risk factors (e.g. intrinsic and extrinsic risks) that need to be targeted for prevention, as well as to monitor the effectiveness of implemented interventions [4–8]. It follows that accuracy and consistency in the calculation and reporting of injury incidence, upon which these priorities are based, is crucial.

Consensus statements for injury surveillance in some sports [9–11] have been published in an effort to guide the accuracy and consistency of injury reporting sought across studies. Methodological papers have also been published with clear definitions and explanations of the different sports injury epidemiological terms [12] and how to interpret or apply them [6, 7, 13, 14]. In short, to facilitate comparison across different sports, settings and follow-up periods, a common measure used to describe the frequency of injury is the incidence proportion, which is essentially the number of new injuries sustained in a defined population (inclusive of the injured person) over a specified period of time. In a sports-injury context, the numerator is generally the number of injuries or number of injured athletes, while the denominator/time component is often reported as the total number of athletic exposures or hours played during the follow-up period.

One of the major challenges in sports injury research is that many athletes experience multiple injuries, therefore contributing to the numerator of injury rates more than once [7]. Where more than one injury is experienced, the terms ‘index injury’ for the first injury and ‘subsequent injury’ for injuries that follow can be used to differentiate injuries in a time-ordered sequence [15]. Where the same body part is injured repeatedly, and is classified as having the same nature, injuries are commonly referred to as being ‘recurrent’ [11, 15–17]. Some subsequent injuries will have a clear biomechanical relation to an initial index injury (e.g. recurrent left-side ankle sprains), while others may be indirectly linked (e.g. calf injury leads to an ankle sprain). Subsequent injuries may also occur due to situational relationships (e.g. smaller player continues to collide with taller, heavier opposition player), or there may be no identifiable relationship to the initial index injury.

The delineation of multiple injuries is vital to the accuracy of determining injury incidence, with incorrect estimates arising if statistical dependencies across injuries are not properly accounted for [7, 18, 19]. The number of (subsequent) injuries is dependent on how injuries are defined (e.g. a new injury, a recurrent injury, first injury, etc.), the method of injury registration (e.g. self-reported, clinical diagnosis), data collection approach (e.g. prospective or retrospective), and length of follow-up (e.g. one season/year only or continued data collection, with longer follow-up having a higher likelihood of more than one injury). Most prospective injury studies have limited their data collection to one sports season only, although it is likely that injury incidence varies over seasons [19]. Moreover, it is possible that injury occurrences across seasons are related to injuries in an earlier season [15, 19]. The risk of injury is generally considered to be higher in people who have had a previous injury, with reasons thought to be related to residual tissue weaknesses, the athlete’s sport, position or behaviour presenting an inherent risk, or the individual returning to sport before complete recovery of an injury [15, 20]. Increased sporting experience has also been shown to reduce injury risk, perhaps owing to better developed physical conditioning or maturity in match play [21, 22]. What is most clear is that there is likely to be an altered risk of future injury over time, particularly for previously injured athletes [19].

The aim of this systematic review is to consider how multiple injuries sustained by individuals have been defined and reported in prospective, long-term (more than one consecutive year or season), injury incidence studies. The review is focused on team ball sports as these sports are often prioritised for injury prevention globally due to large numbers of injuries and participants, and there is a substantial body of literature reporting injury incidence. With the information extracted from the selected studies, we describe whether, and how, multiple injuries within individuals have been addressed and reported. This is important because weaknesses in existing research need to be identified so that they can be addressed in future work through better study design, improved methodological considerations, enhancements to statistical analysis and reporting of injury data or refocussed clinician and researcher training.

## Methods

### Search

A search of the PubMed, Web of Science and Embase databases was performed, on the basis that the leading sports medicine journals are indexed within these databases. The full search strategy is described in electronic supplementary material Appendix S1. Studies that reported injury incidence in team ball sports for more than 1 calendar year or at least two consecutive seasons were included. As not all studies include keywords that consider duration of the study (i.e. we sought more than one season), the search strategy was initially kept broad and a relatively large number of publications screened to allow a specific check of inclusion dates in the full text. All articles were screened by two authors independently, using the criteria described below. Differences were discussed and when no consensus was obtained upon study inclusion, a third author adjudicated. No date restriction was placed for the beginning of the search, with studies included up to an end date of 31 December 2015. Where we could not locate an original paper from our resources, authors were directly contacted.

### Study Selection

Articles were included if (1) they reported injury incidence (i.e. not specific to one injury type) over the time period (e.g. not game injuries only) because we were interested in multiple injuries, studies focusing on single injury types or those sustained in games only were deemed overly restrictive; (2) the study was conducted with participants of team ball sports; (3) data were collected prospectively; (4) the follow-up was more than 1 calendar year or over at least two consecutive seasons; (5) data were collected in a defined cohort (e.g. club, division, school, team, league); and (6) an individual identifier was clear in the collection of data (i.e. an ID or name that would allow the research team to potentially link more than one injury within individuals). The latter criterion was deemed crucial if the original authors had been able to report multiple injuries within an individual.

Non-English language articles, conference abstracts, commentaries and reviews were excluded. Studies based on hospital data were excluded because generally people do not always attend hospital with a sports injury, nor do they exclusively attend the same hospital, therefore such data cannot be used to confidently identify multiple injuries within individuals. Studies conducted during (multiple or consecutive) tournament-style competitions were also excluded as there are often long time frames between such competitions and athletes are likely to participate in other competitions/events over the period during which their injury risk exposures and outcomes would be unaccounted. Where more than one publication was presented from the same source study/dataset, we initially included all publications identified (that met all inclusion criteria) as different approaches may have been used to report/analyse data across papers; if the same data and analysis were reported (albeit for a different aim), only the earliest study published was kept and is presented with a note indicating subsequent publications. The exact number of publications excluded by different reasons is not provided as they were excluded on the first identified reason only (therein potentially presenting an inaccurate picture, as additional reasons, other than the first identified, could also apply).

### Data Extraction

The following data were extracted from the articles, by two authors, into an Excel database. Relevant results were tabulated and presented, along with their descriptive information across five tables.

Descriptive information provided in Table 1 included:

**Table 1:** Methods for defining and reporting sports injuries in studies of more than one consecutive year/season

References	Sport	Number of athletes included	Setting, sex (age range)	Country/region	Study duration, years	Primary focus of injury measure	Sports incapacity	Sports injury	Sports trauma	Multiple injury results
Dagiau et al. [51]	American football	54	College, male (NS)	USA	2 seasons, 1976 and 1977	Risk				
Canale et al. [52]	American football	265	College, male (NS)	USA	5 seasons, 1975–1979	Incidence	Y			Y
Zelisko et al. [53]	Basketball	30	Elite/professional, male and female (NS, adult)	USA	2 seasons, NS	Incidence		Y		
Orchard et al. [54]	Australian football	NS	Elite/professional, senior and junior, male (16–35 and 15–18 years)	Australia	3 seasons, 1992–1994	Incidence	Y	Y		
Hawkins and Fuller [55]	Association football (soccer)	Total NS (average 138)	Elite/professional, male (NS, adult)	UK	NS, 1994–1997	Risk	Y			Y
Powell and Barber-Foss [56]	Mixed (American football, basketball, Association football, volleyball)	52,439 player-seasons^a^	High school, male and female (NS)	USA	3 seasons, 1995–1997	Incidence and risk	Y	Y		Y
Meeuwisse et al. [57]	Canadian football	981	University, male (NS)	Canada	5 seasons, 1993–1997	Incidence	Y	Y		
Powell and Barber-Foss [58]	Mixed (basketball, Association football, baseball/softball)	27,095 player-seasons^a^	High school, male and female (NS)	USA	3 seasons, 1995–1997	Risk	Y	Y		Y
Starkey [59]	Basketball	1094	Elite/professional, male (18–43 years)	USA	10 seasons, 1998–1997	Incidence	Y	Y		
Gunnoe et al. [60]	American football	331	High school, male (NS, grades 8–12)	USA	2 seasons, 1995 and 1996	Risk	Y	Y		Y
Hawkins et al. [61]	Association football	2376	Elite/professional, male (17–35+ years)	UK	2 years, 1997–1998 and 1998–1999	Incidence	Y			Y
Watson [62]	Mixed (Association football, Gaelic football, hurling)	86	County [good club level], male (24 years, ±3.7)	Ireland	2 years, NS	Risk	Y			
Drawer and Fuller [63]	Association football	138	Elite/professional, male (NS, adult and youth)	UK	3 seasons, 1994–1997	Risk	Y			Y
Orchard and Seward [64]	Australian football	2672 player-seasons	Elite/professional, male (NS, adult)	Australia	4 seasons, 1997–2000	Incidence	Y			Y
Gabbett [65]	Rugby league	156	Semi-professional, male (NS, U19 to adult)	Australia	2 seasons, 2000 and 2001	Incidence		Y		
Meeuwisse et al. [66]	Basketball	312	College, male (NS)	Canada	2 years, NS	Incidence and risk	Y	Y		
Turbeville et al. [37]	American football	646	Middle school, male (10–16 years)	USA	2 seasons, 1998 and 1999	Incidence and risk	Y	Y		Y
Turbeville et al. [29]	American football	717	High school, male (13–19 years)	USA	2 seasons, 1998 and 1999	Incidence and risk	Y	Y		Y
McManus et al. [67]	Australian football	535	Community, male (16–50 years)	Australia	2 years, 2000–2001 and 2001–2002	Incidence	Y	Y		Y
Powell and Dompier [68]	Mixed (basketball, American football, Association football, volleyball)	2,358,197 athletic exposures	College, male and female (NS)	USA	2 seasons, 1999–2000 and 2000–2001	Incidence	Y	Y		
Price et al. [69]	Association football	4773	Youth academies, male (9–19 years)	UK	2 seasons, 1997 and 1998	Incidence and risk	Y			Y
Giza et al. [70]	Association football	202	Elite/professional, female (NS, adult)	USA	2 seasons, 2001 and 2002	Incidence		Y		
Kucera et al. [48]	Association football	1483	Elite youth, male and female (9–18 years)	USA	4 seasons, 1997–2000	Incidence and risk	Y			
Hägglund et al. [36]	Association football	197^b^	Elite/professional, male (17–38 years)	Sweden	2 seasons, 2001 and 2002	Incidence	Y			Y
Le Gall et al. [71]	Association football	528	Elite/professional, junior male (U14–U16 years^c^)	France	10 seasons, August 1993–June 2003	Incidence	Y			Y
McManus et al. [72]	Netball	368	Community, female (16–30 years)	Australia	2 seasons, 1997 and 1998	Incidence	Y	Y		Y
Merron et al. [73]	Association football	197	Elite/professional senior and junior, male (18+ years and 16–18 years)	UK	4 years, NS	Incidence	Y			
Ortega-Gallo et al. [31]	Association football	41	Elite/professional juniors, male (mean 23.4 years, ±3.7)	Argentina	2 seasons, 2000 and 2001	More than one injury	Y	Y		Y
Ramirez et al. [25]	American football	5118	High school, male (13–18+ years)	USA	2 seasons, 2001 and 2002	Incidence	Y	Y		Y
Deehan et al. [30]	Association football	210	Elite/professional junior, male (9–18 years)	UK	5 years, 1999–2004	Incidence	Y			Y
Dompier et al. [35]	American football	779	Community junior, male (9–14 years)	USA	2 seasons, 2002 and 2003	Incidence	Y	Y		Y
Rauh et al. [28]	Mixed (basketball, soccer, volleyball)^a^	25,187 player-seasons	High school, females (NS)	USA	3 years, 1995–1997	More than one injury	Y	Y		Y
Brooks et al. [74]	Rugby union	502	Elite/professional, male (NS, adult)	UK	2 seasons, 2002–2003 and 2003–2004	Risk	Y			
Le Gall et al. [27]	Association football	119	Elite/professional junior, female (15–19 years)	France	8 seasons, 1998–2006	Incidence	Y			Y
Johnson et al. [75]	Association football	292	Elite/professional juniors, male (9–16 years)	UK	6 years, 2001–2007	Risk	–	–	–	Y
Knowles et al. [76]	American football	3323	High school, male (14–19 years)	USA	3 seasons, 1996–1999	Incidence and risk	Y	Y		
Drakos et al. [26]	Basketball	1643	Elite/professional, male (17– 43 years)	USA	17 seasons, 1988–1989 to 2004–2005	Incidence	Y	Y		
Dupont et al. [34]	Association football	32	Elite/professional, male (mean 25.6 years, ±3.8)	UK	2 seasons, 2007–2008 and 2008–2009	Incidence and risk	Y			Y
Dauty and Collon [77]	Association football	173	Elite/professional, male (mean 24.1 years, ±4)	France	15 seasons, 1995–1996 to 2009–2010	Incidence	Y			
Mallo et al. [39]	Association football	22 per year	Sub-elite, male (mean 24.8 years, ±3.5)	Spain	4 seasons, 2003–2004 to 2006–2007	Incidence	Y			Y
Mallo and Dellal [78]	Association football	35	Elite/professional, male (mean 21.4 years, ±2.4)	Spain	2 seasons, 2007–2008 and 2008–2009	Incidence and risk	Y			Y
Murphy et al. [79]	Gaelic football	851	Elite/professional, male (mean 24.9 years, 18–36 years)	Ireland	4 seasons, 2007–2010	Incidence	Y			Y
Chalmers et al. [80]	Australian football	382	Sub-elite U18, male (mean 17.1 years, ±0.8, 14–19 years)	Australia	2 seasons, 2010 and 2011	Incidence and risk	Y			
Eirale et al. [81]	Association football	527	Elite/professional, male (mean 26.8 years, ±4.9)	Qatar	3 seasons, 2008–2011	Incidence and risk	Y			Y
Ekstrand et al. [82]	Association football	1743	Elite/professional, male (NS, adult)	Europe	11 seasons, 2001–2012	Incidence	Y			Y
Grooms et al. [83]	Association football	41	College, male (mean 20.1 years, ±2.0, 18–25 years)	USA	2 seasons, 2009 and 2010	Incidence and risk	Y	Y		
Hägglund et al. [1]	Association football	24 teams	Elite/professional, male (NS, adult)	Europe	11 seasons, 2001–2012	Incidence	Y			
Malisoux et al. [84]	Mixed (volleyball, basketball, handball, Association football)	372^d^	Elite youth, male and female (12–19 years)	Luxembourg	3 years, 2008–2011	Incidence	Y			Y
Peck et al. [85]	Rugby (union and sevens)	369	College, military students, male and female(NS)	USA	5 years, 2006–2007 to 2010–2011	Incidence		Y		
Tourny et al. [86]	Association football	412	Elite youth, sex NS (12–20 years)	France	3 seasons, 2009–2011	Incidence and risk		Y		
Barber-Foss et al. [38]	Mixed (basketball, soccer, volleyball)	268	Middle school, female (NS)	USA	3 seasons, 2009–2010	Incidence	Y	Y		
Barron et al. [87]	American football	1295	Community youth team, sex NS, (NS)	USA	5 years, 2000–2004 and 2005	Incidence	Y	Y		
Bjørneboe et al. [88]	Association football	11–14 teams/year	Elite/professional, male (NS, adult)	Norway	6 years, 2002–2007	Incidence	Y			Y
Mohib et al. [89]	Association football	196	Elite youth, male and female (13–17 years)	Canada	4 years, 2008–2012	Incidence	Y	Y		
van der Sluis et al. [33]^e^	Association football	26	Elite junior, males (mean 11.9 years, ±0.84)	Netherlands	3 years, 2002–2007	Incidence and risk			Y	Y
Carling et al. [90]	Association football	59	Elite/professional, male (NS)	France/international	5 seasons, 2009–10 to 2013–14	Incidence and risk	Y			Y
Carling et al. [24]	Association football	14	Elite/professional, male (NS)	France	6 seasons, 2009–2015	Risk	Y			Y
Dompier et al. [91]	Mixed (basketball, soccer, volleyball, American football)	5,146,355 athlete exposures	High school, male and female (NS)	USA	3 years 2011–12 to 2013–14	Incidence	Y	Y		
Ekegren et al. [92]	Australian football	Year 1: 1205 Year 2: 823	Community, male (injured, mean 24 years, SD 4, range 18–41 years)	Australia	2 seasons, 2012 and 2013	Incidence			Y	Y
Gastin et al. [93]	Australian football	69	Elite/professional, male (mean 22.8 years, ±3.6; range 17–32 years)	Australia	4 seasons, 2007–2010	Risk	Y			Y
Kerr et al. [94]	American football	3167	Youth, sex NS (mean 10.7 years, SD 1.9, 5–14.9 years)	USA	2 seasons, 2012 and 2013	Risk	Y	Y		
Kristenson et al. [95]	Association football	2 clubs	Elite/professional, male (NS)	Sweden/Norway	2 seasons, 2008 and 2009	Incidence	Y			
Laux et al. [96]	Association football	22	Elite/professional, male (mean 25.8 years, ±5)	Germany	2 seasons, 2009–10 and 2010–11	Risk	Y			
Lawrence et al. [97]	American football	984 team games	Elite/professional, male (NS)	USA	2 years, 2012–13 and 2013–14	Incidence	Y			
Massidda et al. [98]	Association football	54	Elite/professional, male (mean 25.9 years, ±4.3)	Italy	4 seasons, 2009–2013	Incidence	Y			
Owen et al. [99]	Association football	23	Elite/professional, male (mean 26.8 years, ±4.6, range 18–38)	NS	2 seasons, NS	Incidence and risk	Y			
Palmer-Green et al. [49]	Rugby union	472	Elite junior, males (range 16–18 years)	UK	2 seasons, 2006–07 and 2007–08	Incidence	Y			Y
Pastor et al. [32]	Volleyball	34	Professional, male (mean 25.43 years)	Germany	6 years, 2007–2008 to 2012–13	Incidence and risk		Y		Y
Reeser et al. [100]	Volleyball	637,786 athletic exposures 339,753 athletic exposures	High school and college, female (NS)	USA	4 years, 2005–2006 to 2008–2009	Incidence	Y			
Williams et al. [101]	Rugby union	1462	Elite/professional, male (NS)	England	7 seasons, 2006–2007 to 2012–2013	Incidence	Y			
Hulin et al. [102]	Rugby league	53	Elite/professional, male (mean 23.4 years, SD 3.5)	Australia	2 seasons, NS	Risk	Y			

*NS* not stated, *Y* yes, *SD* standard deviation, U14 = players younger than 14 years, U15 = players younger than 15 years, U16 = players younger than 16 years, U18 = players younger than 18 years, U19 = players younger than 19 years

^a^ Data relating to team ball sports only were extracted

^b^ Participants from both years of the study only

^c^ Age groups not age identified

^d^ Unable to differentiate number of team ball sport participants and other sports

^e^ A 2015 study with the same data was identified and was therefore not included

- participant demographics (sex, age) and sport setting (sport, level of play, country/region)
- number of included athletes (or athlete-seasons or alternative)
- study duration and time frames
- study aim, summarised as the primary focus of the injury measure, being:
  - incidence (number of injuries occurring in the population over time)
  - risk (investigations of independent variables that contributed to the occurrence of the injury)
  - incidence and injury risk in combination (with each defined in the same manner as incidence and risk separately)
  - more than one injury (study was specifically looking at relationships between more than one individual injury occurrence)
- injury definition, coded according to Timpka et al. [23] as
  - sports incapacity—performance/participation impacted on (commonly includes ‘time loss injuries’)
  - sports injury—clinically observed injuries
  - sports trauma—self-reported injuries by athletes
- identification of multiple individual injuries in results (reported within the publication—yes or no)
- severity or duration of injury where relevant to the definition, i.e. sport incapacity with 24-h time loss or the minimum number of games missed.

From publications that presented pooled (grouped) injury results, the number of injuries and number of injured participants were summarised, demonstrating that there was more than one injury occurrence by individuals in these studies (Table 2). Where injury data were presented on a per-person basis (i.e. not only presenting pooled injury results for an entire group), additional information was extracted on how individual injury data were presented, the number of injuries, and the number of individuals injured (Tables 3, 4, 5).

**Table 2:** Number of injuries, athletes and injured athletes in studies that did not specifically report multiple injury occurrences

References	Number of injuries reported	Number of athletes included	Injured athletes (*n* or %)	Number of injuries greater than the number of included athletes	Number of injuries greater than the number of injured athletes
Dagiau et al. [51]	129	54	Not reported	Y	–
Zelisko et al. [53]	272	30	Not reported	Y	–
Orchard et al. [54]	4065	NS	Not reported	–	–
Meeuwisse et al. [57]	1811 injury-events (1971 distinct injuries)	981	Range 53.5–60.4 % (different years)	Y	–
Starkey [59]	7449 athletic-related injuries (9904 inclusive of all reported injuries/illnesses)	1094	961 (all reported injury/illness)	Y	Y
Watson [62]	NA (mean days of injury reported)	86	Not reported	–	–
Gabbett [65]	2253	156	Not reported	Y	–
Meeuwisse et al. [66]	215	312	142	N	Y
Powell and Dompier [68]	68,497	2,358,197 athletic exposures	Not reported	N	–
Giza et al. [70]	173	202	110	Y	Y
Kucera et al. [48]	787	1483	40.7 %	N	N
Merron et al. [73]	427	197	195	Y	Y
Brooks et al. [74]	1475	502	Not reported	Y	–
Knowles et al. [76]	1238	3323	1064	N	Y
Drakos et al. [26]	12,594	1643	Not reported	Y	–
Dauty and Collon [77]	903	173	Not reported	Y	–
Chalmers et al. [80]	232	382	Not reported	N	–
Grooms et al. [83]	17	41	Not reported	N	–
Hägglund et al. [1]	7792	24 teams	Not reported	–	–
Peck et al. [85]	659	369	222	Y	Y
Tourny et al. [86]	618	412	Not reported	Y	–
Barber-Foss et al. [38]	134	268	Not reported	N	–
Barron et al. [87]	694	1295	Not reported	N	–
Mohib et al. [89]	733	196	Not reported	Y	–
Dompier et al. [91]	47,014	5,146,355 athlete exposures	Not reported	–	–
Kerr et al. [94]	1475	3167	915	N	Y
Kristenson et al. [95]	323	2 clubs	Not reported	–	–
Laux et al. [96]	44	22	Not reported	Y	–
Lawrence et al. [97]	4284	984 team games	1172	–	Y
Massidda et al. [98]	NA (mean injury incidence by subgroups reported)	54	Not reported	–	–
Owen et al. [99]	119	23	Not reported	Y	–
Reeser et al. [100]	792 (high school) 1380 (college)	637,786 athletic exposures 339,753 athletic exposures	Not reported	–	–
Williams et al. [101]	6967	1462	Not reported	Y	–
Hulin et al. [102]	205	53	Not reported	Y	–

*Y* yes, *N* no, *NS* not stated, *NA* not applicable, – indicates not able to be answered by data presented in study

**Table 3:** Proportion of athletes with recurrent injuries (same type, same site) identified in studies reporting injury recurrence (*n* *=* 20 studies)

References	Follow-up (years)	Sports incapacity definition (duration of incapacity/injury inclusion)^a^	Recovery definition	% of injuries classified as ‘recurrent/re-injuries’
Recurrence definition within 2 months of initial injury				
Ekstrand et al. [82]	11	Sports incapacity (next match/training)	Return to sport	12
Le Gall et al. [71]	10	Sports incapacity (>48 h)	Return to sport	3
Le Gall et al. [27]	8	Sports incapacity (at least 1 day after day of onset)	Not addressed	4
Carling et al. [90]	5	Sports incapacity (following session)	Return to sport	17^b^
Mallo et al. [39]	4	Sports incapacity (next match/training)	Return to sport	9
Eirale et al. [81]	3	Sports incapacity (next match/training)	Return to sport	12
Recurrence definition >2 months or not specified				
Bjørneboe et al. [88]	6	Sports incapacity (at least 1 day)	Return to sport	20
Hawkins and Fuller [55]	4	Sports incapacity (at least 1 day, not including day of injury)	Days missed	22
Orchard and Seward [64]	4	Sports incapacity (at least one regular match)	Return to sport	17
Murphy et al. [79]	4	Sports incapacity (at least 24 h from midnight on day of injury)	Return to sport	23
Gastin et al. [93]	4	Sports incapacity (missed subsequent game)	Not addressed	28
Powell and Barber-Foss [56]	3	Sports incapacity (any duration), or sports injury (fractures, dental injury) or sports incapacity and sports injury (mild brain injury)	Medical clearance for return to sport	10
Powell and Barber-Foss, [58]	3	Sports incapacity (any duration), or sports injury (fractures, dental injury) or sports incapacity and sports injury (mild brain injury)	Medical clearance for return to sport	14 (girls basketball) 10 (boys basketball)
				10 (girls soccer) 8 (boys soccer)
Drawer and Fuller [63]	3	Sports incapacity (at least 1 day)	Not addressed	22
Malisoux et al. [84]	3	Sports incapacity (at least one match/training)	Not addressed	11 (time period 1) 20 (time period 2) 26 (time period 3)
Hawkins et al. [61]	2	Sports incapacity (>48 h, not including day of injury)	Return to sport	7
Price et al. [69]	2	Sports incapacity (>48 h, not including day of injury)	Return to sport	3
Mallo and Dellal [78]	2	Sports incapacity (any part of match/training)	Days missed	15
Ekegren et al. [92]	2	Sports trauma	Days missed	17
Palmer-Green et al. [49]	2	Sports incapacity (at least 24 h from following day)	Days missed	15 (academy) 21 (school)

^a^Definitions from Timpka et al. [47]

^b^Only recurrences sustained while participating in a national team were reported

**Table 4:** Summary of the proportion of athletes with more than one injury (from the total included and injured only) [*n* *=* 8 studies]

References	Follow-up (years)		Number of athletes included	Number of injured athletes	Number of injuries	Number of athletes with more than one injury	% of injured athletes with more than one injury	% of all included athletes with more than one injury	Average number of injuries per included athlete^a^	Average number of injuries per injured athlete^b^
Le Gall et al. [27]	8		119	110	619	99	90	83	5.2	5.6
Rauh et al. [28]										
Basketball	3		–	1271	1748	335	26	–		1.4
Soccer			–	1258	1771	342	27	–		1.4
Volleyball			–	580	701	100	17	–		1.2
McManus et al. [72]	2	1997	368	112	Minimum 146	29	26	8		–
		1998		160	Minimum 258	70	44	19		–
McManus et al. [67]	2		535	400	1031	254	64	47	1.9	2.6
Gunnoe et al. [60]	2		331	121	165	32	26	10	0.5	1.4
Turbeville et al. [29]	2		717	100	132	–	17–26	–	0.2	1.3
Ramirez et al. [25]	2		5118	1307	1700^c^	298	23	6	0.3	1.3
Canale et al. [52]	5		265	227	283	51	22	19	1.1	1.2

^a^Calculated as number of injuries/number of included athletes

^b^Calculated as number of injuries/number of injured athletes

^c^Total injury events (more than one diagnosis given to some events)

**Table 5:** Outcomes of included studies (*n* *=* 11) that have reported the number/mean injuries per athlete over time

References	Outcome
Mean injuries per athlete per season	
Ortega-Gallo et al. [31]	9.5 injuries per athlete per season
Le Gall et al. [71]	2.2 injuries per athlete per season
Deehan et al. [30]	0.6 injuries per athlete per season
Ekegren et al. [92]	0.7 injuries per athlete per season (first and second seasons)
Mean injuries per athlete over study period	
Le Gall et al. [27]	5.2 injuries per athlete
Mallo et al. [39]	3.6 ± 0.7 injuries per athlete
Mallo and Dellal, [78]	2.3 ± 1.8 injuries per athlete
Dupont et al. [34]	5.2 ± 3.7 injuries per athlete
Pastor et al. [32]	1.94 per athlete (acute injuries) 0.64 per athlete (overuse injuries)
van der Sluis et al. [33]	6.85 ± 5.46 for the total group during the 3 years
Number of injuries per athlete per 1000 h	
Johnson et al. [75]	2.23 injuries each athlete per 1000 h

## Results

**Fig. 1:**
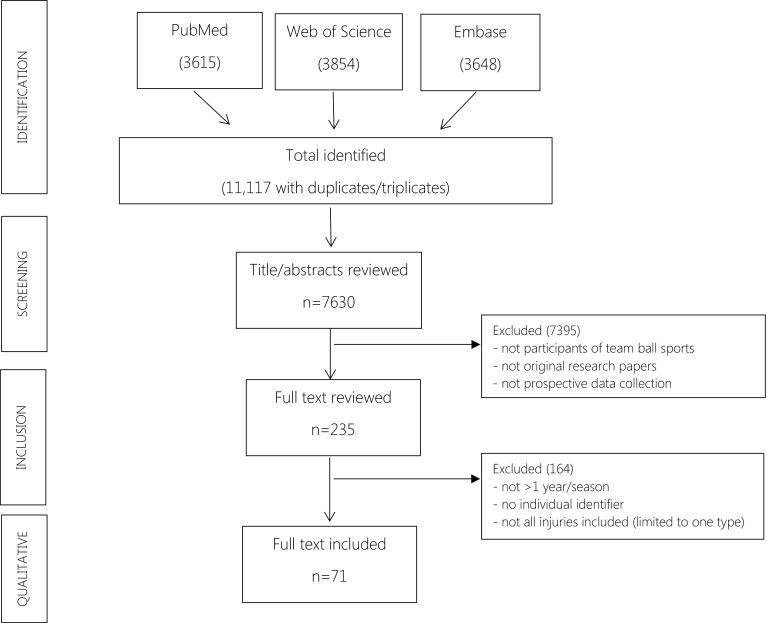
Study selection and inclusion process

After removing duplicates, the search resulted in 7630 studies. The majority of these studies were excluded after reading the abstract, with 235 full-text publications retrieved for detailed reviewed (see Fig. 1). Based on information in the full-text of publications, a further 164 papers were excluded because they had less than 1 year of data collection, collected data at a team level not an individual level, were not full original research papers or did not cover all injuries (e.g. focusing only on match injuries or knee injuries). Overall, 71 publications were retained for analysis.

The studies covered a range of different sports and settings (Table 1), with between 14 [24] and 5118 [25] individual participants reported. Twelve studies presented participant inclusion without individuals, but rather as the number of teams per year or the total number of athlete-seasons combined. Not all participants/teams were followed for the full duration of a study and it was uncommon for authors to report specific follow-up times of individuals/teams. Studies were conducted over a minimum of two seasons/years (in line with our inclusion criteria), to a maximum of 17 consecutive years [26]. Almost one-quarter (*n* *=* 16, 23 %) of included studies were published in 2015 or later.

Summarising the broad aim of the papers, half (*n* *=* 39 of 71, 55 %) were aimed at reporting the incidence of injury, one-quarter (*n* *=* 17, 24 %) reported both injury incidence and injury risk, 18 % (*n* *=* 13) were focused on the risk of injury alone, and 3 % (*n* *=* 2) were aimed at investigating more than one injury occurrence.

Injury definitions were most commonly based around the Timpka et al. [23] domain of sports incapacity (Table 1). Sports incapacity was often used in combination with a specified definition from the sports injuries domain (a clinically observed injury) wherein certain diagnoses (e.g. fractures, dislocations) were included, irrespective of the incapacity incurred. For example, the definition may have read similar to ‘injuries that resulted in over 24 h of time loss, as well as all fractures and dislocations’.

Keeping in mind the inclusion criteria that an individual identifier was clear in the collection of data, almost half (*n* *=* 34 of 71, 48 %) of the studies did not report the number or distribution of multiple individual injuries within the study (Table 2). From these studies, the number of injured athletes could not be confirmed from data in 24 of 34 publications (71 %). The number of injuries exceeded the number of included athletes in 17 of 26 (65 %) papers in which the relevant injury and athlete numbers could be identified. Furthermore, the number of injuries exceeded the number of injured athletes in 8 of 9 papers (89 %) where relevant numbers could be identified. These two observations, in particular, indicate the presence of multiple injuries in some athletes.

Where multiple injuries were reported (*n* *=* 37 of 71, 52 %), studies mostly addressed recurrent injuries only (*n* *=* 20, 28 % of all studies, 59 % of studies reporting multiple injury results) (Table 3). The proportion of recurrent injuries varied from as few as 3 % of injuries to as many as 26 %, and was influenced by the injury definition, duration of the study, and recurrence criterion being within 2 months or greater than 2 months.

Eight studies (11 % of all studies, 22 % of studies reporting multiple injury results) reported a frequency proportion of athletes who had sustained varying numbers of injuries (e.g. up to 8, or grouped as 3+) (Table 4). Results from each of these studies were collapsed to enable comparable presentation of the number and proportion of athletes with more than one injury. As a proportion of all included athletes, as few as 6 % [25] and as many as 83 % [27] of athletes had multiple injuries recorded within the study period. As a proportion of injured athletes, there were as few as 17 % [28, 29] and as many as 90 % of athletes with more than one injury [27].

Eleven studies (15 % of all studies, 30 % of studies reporting multiple injury results) presented the mean number of injuries per athlete for a specified time (Table 5). A range of 0.6 [30] to 9.5 injuries [31] was observed when considered as an average per athlete per season. Looking at the mean per athlete over the whole study period, the range varied from 0.6 to 1.9 (injuries reported separately as overuse and acute, respectively [32]) to 6.9 injuries [33] per athlete over the study period.

In the included publications, other methods used to report multiple individual injuries included the numerical range of injury numbers sustained by an individual (e.g. Pastor et al. [32] and Dupont et al. [34]), the maximum number of injuries sustained by an individual (e.g. Dompier et al. [35]), the risk of injury in subsequent seasons (e.g. Rauh et al. [28] and Hägglund et al. [36]), or simply stating that some athletes had more than one injury during the season [37].

## Discussion

Documenting injury incidence requires consistent and precise measures in order to accurately study risk factors for injury and to prioritise and monitor preventive interventions. Experiencing more than one injury is common in sports settings, and this presents challenges to the collection and reporting of injury incidence data. Different approaches have been used in addressing this challenge. In this review, we aimed to identify the most common data reporting methods for handling cases of multiple injuries in longitudinal studies of team ball sport participants. Our key finding was that in half of all studies that extend for more than one year/season, the number of injuries or injured athletes was pooled (grouped) over the whole time period and reported as an overall total. In other words, the occurrence of multiple injuries was not explicitly reported despite these same studies having collected data at an individual level—one of our inclusion criteria for the review. Of the studies that did report multiple injuries, many were limited to inclusion of recurrent injuries only (i.e. those of the exact same type and at the same site). Very few authors have recorded and reported multiple injuries of differing natures and they have largely done so using inconsistent methods across studies that prevented an in-depth comparison of the results or strong conclusions on the likelihood of athletes experiencing subsequent injury.

### Understanding the Current Limitations in Injury Reporting

**Fig. 2:**
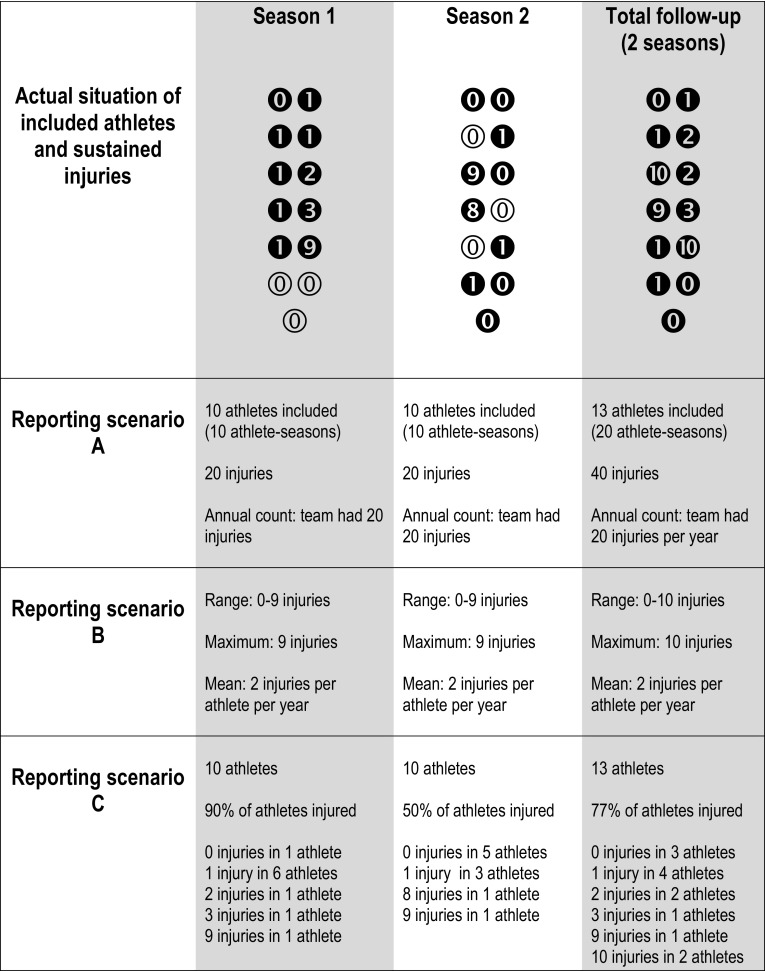
Different reporting scenarios for injuries in a fictional team of 10 athletes followed for two consecutive seasons. *Black circles* indicate that athletes were included in the team during the season, *white circles* indicate that athletes were not included in the team during the season, and the* number in circle* indicates the number of injuries in the specified timeframe

The most common presentation of data was pooled injury counts summarised across teams/seasons. This is problematic if there is no indication of the number of athletes sustaining more than one injury, particularly in studies aiming to infer injury risk. Although many of the included studies did not have a primary aim to look at more than one injury in individuals, the issue still needs to be considered. For example, a common study aim might be to follow specific teams/squads to determine changes in team outcomes based on new training practices. Similarly, school-level sport competitions may be interested in how overall safety levels change over time, not individual risks. However, when reporting injury incidence for an understanding of injury risk, the individual distribution of the number of injuries is important because of its impact on the correct statistical reporting of incidence rates [17]. As an example, one study reported 134 injuries in 268 athletes over 3 years “yielding a risk of injury of 50 %” [38] (p148). Such a statement is only true if there were no athletes with more than one injury within the study population. As the authors did not report the distribution of injuries by participants, it is not possible to verify this but, in general, findings reported in this manner will miss details that are vital for interpreting results. In its most simplistic form, this information is depicted in Fig. 2, reporting scenario A. It can be seen that the incidence outcomes within this scenario (10 athletes included, with 20 injuries in total) are similar for both seasons, although the actual situation is very different (refer to the numbers in *black circles*).

Where the potential for an individual having more than one injury in the results was recognised, this was sometimes addressed by reporting the range of injuries sustained or the maximum number of injuries sustained by an individual, e.g. ‘between one and *n* injuries occurred’. Other authors acknowledged the problem of multiple injuries by reporting the mean and standard deviation for injuries per athlete/team per season (e.g. “On average, an athlete incurred 3.6 ± 0.7 injuries” [39]). Figure 2, reporting scenario B, shows that these approaches still fail to adequately address how an individual with more than one injury impacts the group result as it can again be seen that incidence outcomes using this scenario are similar for both seasons (range 0–9 injuries; maximum: 9; mean: 2 injuries per athlete per year), although the actual situation is very different (refer again to the numbers in black circles).

It was clear that many cohort participants within the reviewed studies had likely experienced more than one injury (i.e. where the number of injuries recorded exceeded the number of injured participants). This is common in studies of sports injury. Similarly, the number of injured athletes is often less than the number of athletes included, as can be seen in Table 2. In other words, injury data in team sports are commonly skewed, with a large number of zero counts, and this distribution needs to be considered [17]. One, or just a few athletes, might sustain the majority of team injuries [15, 28], as shown in Fig. 2, scenario C. For any team, at any time point, the proportion of athletes contributing to the injury count will differ. Therefore, even those studies reporting team-level data and overall changes in annual injury counts (and not necessarily concerned by any individual outcomes) still need to consider individual bias in the results.

Irrespective of whether a simple or complex study design and analysis method is chosen to meet a study’s aim, authors must clearly identify the relevant details of included variable characteristics as they relate to multiple injuries. As a minimum, the frequency distribution of the number of injuries is recommended for inclusion in future work (see example in Finch and Cook [15]). This information would at least enable readers to gain a sense of the individual burden and scope for potential dependency between multiple injuries sustained, as well as providing crucial support for the choice of statistical modelling applied (e.g. if there is overdispersion of data or a high number of zero counts) [17, 40]. This more detailed presentation of the data is similar to recommendations of analyses where time to injury data is considered, with improved reporting of assumptions and detailing of the event being modelled [7, 41].

### Other Findings Identified from the Review

A key finding was that the majority of studies presented broad annual injury rates across teams/seasons. Few gave specific reasoning as to why the results were presented in this way, with the exception of an aim being to compare year-to-year outcomes. One study team reported that results in two of their publications were both pooled as there was no statistically significant change over time [29, 37]. Reasons why pooled data may be favoured by researchers could include the avoidance of difficulties with ethical approval if data are reported at a group level not an individual level; a need for confidentiality, as pooling the data hides small or distinctive values; the analysis is less complicated if data are grouped; pooling data will give larger numbers, allowing for more sophisticated analyses; or to avoid the difficulties in differentiating between index/recurrent/subsequent injuries. While the results across studies were presented largely as pooled data at the team level, the collection of data itself was at an individual level, with some form of identifier in the data collection process a requirement of our study inclusion process. Based on these studies, it would seem that it is easier to record, rather than adequately report, more than one injury within study cohort members.

The overall number of publications extending beyond one season/year has increased over the last 2–3 years. This possibly reflects a growing understanding of the need to look not only beyond one season when considering injury risk but also new validated technology to facilitate long-term data collection. For example, recent studies document the value of online applications and SMS text messaging in enabling a relative ease of access and follow-up of participants [42, 43]. Online and automated forms of data collection will be further enhanced in coming years if analytical methods and reporting algorithms that make the most of individual-level data also become widely used.

A limitation of the current understanding about multiple injuries in the reviewed studies is the focus being largely limited to recurrent injuries—injuries that are exactly the same site and same type as previously incurred by an individual. Clinical experience and new classification models substantiate the need to look at the relationships between injuries more broadly [15]. Another barrier to reporting multiple injuries is how to document and measure recovery. Recovery is a key element in determining whether a subsequent injury is new or potentially related to a previous occurrence. Unlike other injury contexts, the risk of more than one injury in the sports setting is high and there can be a short time lag between the injury events. Within this review, we have not identified and reported how recovery was defined or addressed, although the importance of this topic and its relation to reporting multiple injuries is acknowledged. We initially attempted to include the information but found the message confused our primary aim of reporting methods of multiple injuries. What was clear in terms of recovery was that authors mostly used a return-to-sport definition, with or without clearance from a medical specialist and at differing levels (i.e. training, partial return to competition, or full return to competition). Operationalising ‘return to sport’ as an outcome measure is challenging [44, 45] and, indeed, there is an entire consensus statement developed for this specific issue [46]. With the exception of the date of return, recovery from injury as a clearly defined outcome currently lacks universal objective measures and is influenced by a range of potential factors, including access to medical care and previously sustained injuries [44–46]. A second challenge in defining recovery arises with the recent expansion of more inclusive injury definitions that are no longer consistent with earlier recovery definitions. For example, if a holistic approach is used in a definition, such as ‘recordable incidents inclusive of psychological factors’[47], then it is not clear how concepts of readiness, fear and confidence contribute to recovery and return-to-play definitions. The ongoing discussion among researchers, and proposed methods for addressing this topic, are promising and will only serve to improve not only an understanding of recovery but also, in turn, multiple injuries.

### Limitations of the Review

We included all studies where it was clearly stated that an individual unique code had been assigned to identify each included athlete, in which case an individual injury history could theoretically have been traced over the duration of the study. Despite a conservative approach to excluding papers, referring to the full text for information in order to be certain of exclusions, there remains a possibility of having missed papers owing to the challenges of identifying and interpreting authors’ methods for inclusion and recording of injury data. In particular, it was often unclear from the identified studies whether individual data were available based on the described methods of data collection. Nevertheless, it is likely we have captured a highly representative sample of the majority of publications that have adopted the most commonly used methods in the general team ball sports injury research literature. Future work could consider multiple injuries in individual sports and determine whether reporting in this setting is different. It would also be worth assessing if there is better or worse capture of overuse injuries than the more traumatic, time-loss injuries, which tend to feature more heavily in team ball sport research, and determining whether this is due to data collection methods or true prevalence. Although the inclusion criteria specified that all injury types were to be captured by the studies (i.e. excluding studies of one injury type only), the delineation between injury types (traumatic, overuse) was not the focus of our results. As our conclusions are in relation to the reporting of injury counts only, they will apply equally to different injury types and also to non-team ball sports.

We chose to limit our studies to those of more than one season/year duration as an increased number of injuries is likely over increased time and we were interested in how researchers handled this methodologically. However, with this length of time it was not always clear how many individuals were followed. It is possible that in some studies, none, or very few individuals were followed up, as was the case with statements such as “only 45 players (7 %) were present in both years” [37] (p. 277). In some instances, authors reported the number of people who played each of the seasons whether or not they were injured, information that would be valuable to include in future studies. As examples, one study reported the number of athletes for each subsequent season as “thirty six players were followed for one season, 163 for two seasons, 701 for three seasons, 412 for four seasons and 171 for five or six seasons” [48] (p. 464). Similarly, in other papers, authors reported “the average player was in the database for 3.7 ± 3.2 seasons” [26] (p. 286) and 56 of 472 (12 %) athletes were included for the full duration of the study [49]. Our study findings are thus limited by the data reported in the original studies as we cannot be sure how many individuals were followed-up for a given length of time.

## Conclusions

Limitations associated with the data that have been reported in previous sports-injury epidemiological studies have significantly hindered the ability to provide robust evidence about subsequent injuries. While differences in wording and definitions used for injuries, recovery, exacerbation, recurrence and so forth may seem inconsequential at times, their influence is far reaching [50], with differences affecting the accuracy of both the classification of injuries and calculation of injury incidence. This in turn limits our understanding of risk factors for injury.

Sports medicine research is not far from realising the collection and analysis of data that identify individual multiple injury occurrences, although the gap that remains may be challenging to bridge. Collaboration between different professions (clinicians, epidemiologists and biostatisticians) early in the design of a study will help to address some of these challenges. Ultimately, injury prevention efforts rely on accurate incidence estimates, and ongoing developments in this area are encouraged in order to advance understanding of the causal underpinnings of sports injuries and their prevention.

## Electronic supplementary material

Below is the link to the electronic supplementary material.

Supplementary material 1 (DOCX 10 kb)
