# Supplementary material for: Reporting Multiple Individual Injuries in Studies of Team Ball Sports: A Systematic Review of Current Practice
Source: Sports Med. 2016 Oct 26;47(6):1103–22. doi: 10.1007/s40279-016-0637-3 (PMC5432578; doi:10.1007/s40279-016-0637-3)
Supplement: Supplementary file 1 — Supplementary material 1 (DOCX 10 kb) [file 40279_2016_637_MOESM1_ESM.docx]

**Electronic Supplementary Material Appendix S1**

**Pubmed**

(recurrent OR recurrence OR multiple OR subsequent OR repeat OR repetitive OR epidemiolog* OR incidence OR longitudinal OR prospective OR rate OR count OR frequency)

AND

(injur*)

AND

(“Team sport” OR “Team sports” OR Soccer OR Football OR Rugby OR Basketball OR Netball OR Handball OR Volleyball OR Gridiron OR “ball sport” OR “ball sports”)

**Web of Science**

((recurrent OR recurrence OR multiple OR subsequent OR repeat OR repetitive OR epidemiolog* OR incidence OR longitudinal OR prospective OR rate OR count OR frequency) AND (injur*) AND (Team sport OR Team sports OR Soccer OR Football OR Rugby OR Basketball OR Netball OR Handball OR Volleyball OR Gridiron OR ball sport OR ball sports))

**Embase**

(‘recurrent’ OR ‘recurrence’ OR ‘multiple’ OR ‘subsequent’ OR ‘repeat’ OR ‘repetitive’ OR epidemiolog* OR ‘incidence’ OR ‘longitudinal’ OR ‘prospective’ OR ‘rate’ OR ‘count’ OR ‘frequency’)

AND

(injur*)

AND

(‘Team sport’ OR ‘Team sports’ OR ‘Soccer’ OR ‘Football’ OR ‘Rugby’ OR ‘Basketball’ OR ‘Netball’ OR ‘Handball’ OR ‘Volleyball’ OR ‘Gridiron’ OR ‘ball sport’ OR ‘ball sports’)
